# The risks of learning: confounding detection and demographic trend when using count-based indices for population monitoring

**DOI:** 10.1002/ece3.1258

**Published:** 2014-12-02

**Authors:** Vincenzo Gervasi, Henrik Brøseth, Olivier Gimenez, Erlend B Nilsen, John D C Linnell

**Affiliations:** 1 Norwegian Institute for Nature ResearchHøgskoleringen 9, 7034, Trondheim, Norway; 2 Centre d'Ecologie Fonctionnelle et Evolutive, UMR 5175Campus CNRS, 1919 Route de Mende, Montpellier Cedex 5, F-34293, France

**Keywords:** Autocorrelation, citizen science, demographic monitoring, den, detection probability, learning period, nest, population size, population trend, reproduction

## Abstract

Theory recognizes that a treatment of the detection process is required to avoid producing biased estimates of population rate of change. Still, one of three monitoring programmes on animal or plant populations is focused on simply counting individuals or other fixed visible structures, such as natal dens, nests, tree cavities. This type of monitoring design poses concerns about the possibility to respect the assumption of constant detection, as the information acquired in a given year about the spatial distribution of reproductive sites can provide a higher chance to detect the species in subsequent years. We developed an individual-based simulation model, which evaluates how the accumulation of knowledge about the spatial distribution of a population process can affect the accuracy of population growth rate estimates, when using simple count-based indices. Then, we assessed the relative importance of each parameter in affecting monitoring performance. We also present the case of wolverines (*Gulo gulo*) in southern Scandinavia as an example of a monitoring system with an intrinsic tendency to accumulate knowledge and increase detectability. When the occupation of a nest or den is temporally autocorrelated, the monitoring system is prone to increase its knowledge with time. This happens also when there is no intensification in monitoring effort and no change in the monitoring conditions. Such accumulated knowledge is likely to increase detection probability with time and can produce severe bias in the estimation of the rate and direction of population change over time. We recommend that a systematic sampling of the population process under study and an explicit treatment of the underlying detection process should be implemented whenever economic and logistical constraints permit, as failure to include detection probability in the estimation of population growth rate can lead to serious bias and severe consequences for management and conservation.

## Introduction

In its essence, management and conservation of wildlife populations are mostly aimed at affecting the rate and direction of population change over time. In some cases, the negative demographic trend of a threatened species needs to be turned around, in order to prevent its extinction (Butchart et al. 2010). In other instances, such as for the control of biological invasions, a strongly positive growth rate of one or more undesired alien species needs to be limited or reversed (Mack et al. 2000). Also, many natural populations are exploited by humans through harvest, or have the potential to affect other important ecosystem services, such as food crops, timber extraction, livestock, carbon sequestration, human security, etc. (Mace et al. 2012). Therefore, managers often aim at regulating populations, to ensure that the provision of those services can be sustained while balancing wildlife population persistence and human well-being (Murdoch 1994). In all these instances, an accurate and timely assessment of population trends over time is of obvious importance to any adaptive management system, as it provides crucial information on the system's response to human actions (Keith et al. 2011; Bunnefeld et al. 2013).

With respect to “how” the monitoring of population trend is performed, substantial progress has been made over the past decades in terms of sampling design and analytical methodologies. In particular, it has been recognized that a proper treatment of the detection process is required to avoid producing biased estimates of population rate of change (Yoccoz et al. 2001). Distance sampling (Buckland et al. 1993) and capture–recapture (Otis et al. 1978) are two classes of method that can be used to estimate the detection probabilities associated with count statistics, and to produce unbiased estimates of abundance and population rate of change in the face of imperfect detection.

Despite such progress in design and analysis, one of three monitoring programmes on animal or plant populations is still focused on a continuous index (usually counts of individuals), which does not include any formal treatment of the underlying detection process (Marsh and Trenham 2008). The main reported reason for this pattern is the limitation imposed by time shortage or by the lack of economic resources, which prevent the application of more robust, but also data demanding techniques (Danielsen et al. 2003; Marsh and Trenham 2008; Reynolds et al. 2011). This is particularly the case for elusive species, such as terrestrial carnivores or nocturnal birds, whose sampling is affected by a low effectiveness of survey methods and high costs, as a consequence of the species’ behavior, activity, preferred habitats, and low overall densities (McDonald 2004). As a result, quick to obtain and cheaper indices of population trend, derived from cumulative counts of individuals, are still widespread monitoring methods used to inform the decision-making process for management and conservation (Marsh and Trenham 2008), even though they often provide low power in accurately estimating population size and trend (Katzner et al. 2007).

The use of simple cumulative counts of individuals as a tool to monitor population trend is based on the strong assumption that detection probability stays constant over time. If we define true population growth rate 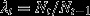, and the estimated population growth rate 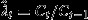, where *N*_*t*_ and *C*_*t*_ are the population size and population count in year *t*, 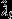 is an unbiased estimate of *λ*_*t*_ only if 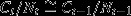, that is, only if detection probability does not change between years. If detection increases with time, the estimator of population growth rate will be positively biased, whereas a negative bias will occur if detection probability decreases between years. Archaux et al. (2012) have shown that even small (4–8%) differences in detectability between two treatments can lead to a 50–90% increase in the risk of erroneously detecting a difference in abundance. Still, despite being a crucial aspect in the application of this type of estimator, the assumption of constant detection is rarely verified or discussed, when reporting the results of monitoring programmes based on count indices. At the most, the statement that sampling effort and monitoring scheme have remained the same over time is used to reassure readers that the ability to detect individuals of the study species has not changed throughout the monitoring period (Yoccoz et al. 2001).

One particular case of count-based population indices refers to the use of fixed visible structures, such as natal dens, nests, tree cavities, etc., to detect the reproductive portion of the population, which is then compared between years to derive estimates of population growth rate. Counts of natal dens, nests, and more generally of reproductive units have been widely used to estimate minimum abundance and population trend of mammals (Wilson et al. 2003; Linnell et al. 2007; Hájková et al. 2008*;* Kindberg et al. 2009; Brøseth et al. 2010), birds (Hatfield et al. 1996; Gilbert et al. 1998; Seamans et al. 2001; Hardey et al. 2006), amphibians (Schmidt 2003), and reptiles (Madsen and Shine 1999).

The use of fixed structures to monitor populations of rare or elusive species has some obvious advantages, as in elusive species dens and nests are often easier to detect than the individuals occupying them. Moreover, in most of the above-cited species, the same denning or nesting location may not only be used by the same individual for several years, but also inherited by other individuals after it has been abandoned. This means that the information acquired after the first species detection at any of the sampling locations increases the chances to detect the same species at the same location in subsequent years, even if sampling effort and design remain unchanged throughout the monitoring period. While providing some advantages, this type of monitoring design also poses strong concerns about the possibility to respect the assumption of constant detection. In fact, in all cases in which any information acquired in a given year about the spatial distribution of reproductive sites can provide a higher chance to detect the species at the same or at a near location in subsequent years, the system is intrinsically prone to a temporal increase in detection probability. In more general terms, whenever the information acquired in year *t* is retained, the population count in year *t +* 1 is no longer a random independent sample of true population size, but it will show a certain degree of temporal correlation with the population counts obtained in all previous years, even if the detection probability of never identified reproductive units remains constant. Also, such accumulation of knowledge about the demographic process under study is expected to be more relevant during the first years of monitoring, during which the negative effects of a trend in detection probability are also expected to be more serious.

The consequences of a temporal trend in detection probability on the performance of count-based demographic monitoring have been extensively treated (Barker and Sauer 1992; Yoccoz et al. 2001) and usually described as an effect of unequal year-to-year sampling effort, habitat characteristics, weather conditions, species behavior, etc. Much less attention has been devoted to assess how a more general accumulation of knowledge can generate an intrinsic increase in the ability to detect a species, even when all the above-described factors remain unchanged through time.

To explore this subject, we developed an individual-based simulation model and evaluated how the accumulation of knowledge about the patterns of a population process (in this case the use of reproduction sites) can affect the accuracy of population growth rate estimates, when using simple count-based indices. We made the model flexible and general enough to apply it to species with different life expectancy, and with a different year-to-year fidelity to reproduction sites, thus mimicking a varying persistence of the information acquired by the monitoring system through time. We also present the case of wolverines (*Gulo gulo*) in southern Scandinavia as an example of a monitoring system with an intrinsic tendency to accumulate knowledge and increase detectability. Finally, we discuss the predictions of our model in terms of its potential contributions to improve the monitoring and management of rare and elusive species.

## Methods

### Simulation model

We built the individual-based model comprising three successive processes as follows: (1) the demography of the study population; (2) the yearly occupation of reproduction sites; and (3) the detection process.

Given the general scope of our study, the species demography was described by a simple, density-independent, exponential model.


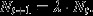


The simulated population comprised only female individuals of reproductive age, that is only those individuals potentially able to occupy a reproductive site. All the individuals in the population at a given time step were included as independent objects in the model, with a binomial probability *ϕ* to survive to the next time step, whereas the number of recruits *R*_*t*_ (the number of new females reaching reproductive age at a given time step) was calculated as follows:


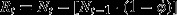


All the individuals alive in the population at a given time step (new recruits + individuals surviving from the previous time step) were assigned a further binomial probability *f* of reproducing. In case of reproduction, each individual was offered the possibility to select a reproduction site among a large number of potential sites (*S*), so that the number of available sites was not a limiting factor in our model. If reproducing for the first time, an individual had two possibilities as follows: (1) to choose a new site; (2) to inherit a site previously occupied by another individual. Such choice was controlled by the probability *h* to inherit a reproduction site. If an individual had already reproduced in previous years, it was bound to occupy the same site, until a maximum number of years, set by the parameter *π*, after which a new site had to be selected. In a biological sense, the parameter *π* represents the maximum number of years a given site can be potentially used for reproduction. It can be affected not only by the physical degradation of the site, such as in the case of a den or a hollow tree, but also by the behavioral ecology of the study species (fidelity to reproduction sites, stability of territories, etc.). In the specific context of our simulations, the parameter *π* represents the number of years during which the information acquired when detecting the study species for the first time at a given site can enhance the ability to redetect the same species in subsequent years. The parameter is hereafter referred to as *site persistence*. Overall, the probability for a new site to be occupied in a given year was described as follows:


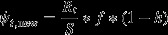


whereas the probabilities for a site occupied in year *t*−1 to be reoccupied in year t were as follows:


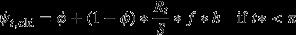



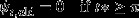


with t* being the number of years since the first time a given site was used.

**Figure 1:**
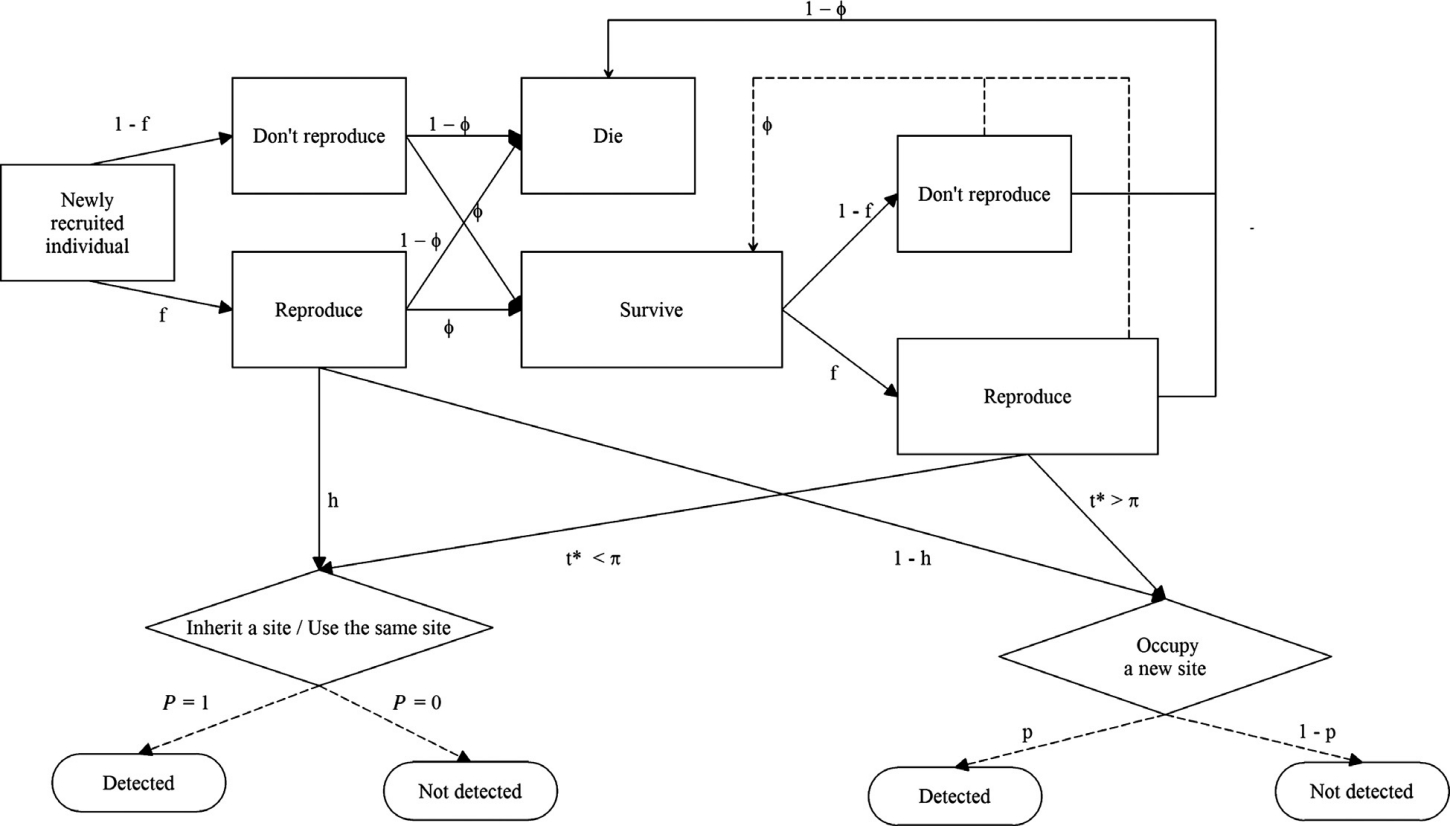
Conceptual diagram describing the structure of the individual-based model, used to explore the potential bias in estimating population growth rate, when using a count-based index. Each individual enters the model when first recruited for reproduction in the population. Transition probabilities are based on the following notation: t*, number of years since the first time a reproduction site has been used; *π*, persistence of a reproduction site (years); *ϕ*, survival probability; *h*, probability for a new individual to inherit a reproduction site; *P*, detection probability; *f*, probability to reproduce.

After simulating both the demographic and site occupation processes in the population, we also included the detection process into the model, which regarded sites and not individuals. Conditional on being occupied, each site was detected with a binomial probability *P*, with the exception of those sites already detected at least once. In that case, detection probability was fixed to one. The complete list of parameters, used to build the individual-based model, is provided in Table 1, whereas the logical structure of the model is shown in Fig. 1.

**Table 1:** Description of parameter symbols and values used in the individual-based model of reproductive site occupation and detection

Symbol	Value/Range/Formula	Description
*N*_*0*_	500	Initial number of females in reproductive age in the population
*λ*	0.95–1.05	Population growth rate
*e*	0–1	Environmental stochasticity (coefficient of variation of *λ*)
*N*_*t*_	*λ* · *N*_*t*−1_	Number of females in reproductive age in the population at time *t*
*ϕ*	0.4 – 0.9	Survival probability
*R*_*t*_	*R*_t_ = *N*_*t*_ – [*N*_*t*−1_ · (1−*ϕ*)]	Number of new recruits each year
*f*	0.5–0.9	Reproduction probability
*h*	0–0.5	Probability to inherit a reproduction site previously occupied by another individual
*π*	0–10	Persistence of a reproduction site (years)
*P*	0.1–0.9	Detection probability of each reproduction site

After completing the detection process, for each time step, we produced estimates of population growth rate, based on the observed count statistics:


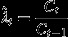


We first ran the model under a set of fixed parameters, to preliminarily assess the sensitivity of population growth estimates to variation in model parameters. In particular, we compared the performance of the model when applied to an increasing vs. decreasing population, on short-lived vs. long-lived species, using ephemeral vs. highly persistent reproduction sites, and for different levels of detection probability. For each scenario, we ran 1000 iterations over 20 time steps from an initial population size of 500 individuals and evaluated the performance of the simulated monitoring system in terms of % relative bias of lambda estimates, and calculating the number of years necessary to stabilize bias to values <0.05, hereafter referred to as the *learning phase*. As environmental stochasticity in population growth rate can strongly affect the ability to detect changes in population size and trend (Lande et al. 2003), we also implemented a modified version of the demographic model, by adding an environmental stochasticity term *e*, which corresponded to the coefficient of variation of population growth rate. We then compared the performance of the count-based index under the deterministic and stochastic models.

After exploring the general characteristics of the model with a scenario-based approach, we performed a more comprehensive sensitivity analysis, allowing all parameters to vary randomly and simultaneously. This allowed us to assess the relative importance of each parameter in the model, by estimating the expected variation in model outputs for a small change in each of the inputs (McCarthy et al. 1995). For each iteration, we extracted parameter values from a uniform distribution, whose range is provided in Table 1. After completing 1000 runs, we fitted a generalized linear model using all the standardized input parameters as predictors, to allow comparison among the effects of each predictor, and chose the length of the learning phase as response variable, using a Poisson distribution. The standardized regression coefficients of each input parameter were used to assess its relative importance in affecting the length of the learning phase. The individual-based model is available through the R (R Development Core Team 2008) function *sim_count* in Appendix S1.

### The wolverine study case

Today around one thousand wolverines live in Scandinavia (Persson and Brøseth 2011), with a continuous distribution which embraces Norway, Sweden, and Finland. Since 1996, the southern portion of this population in Norway and Sweden has been monitored through a simple cumulative count of reproductive units at natal dens. Wolverines usually den in a system of snow tunnels, consisting of a rock cavity or simply of a sheltered slope where snow accumulates (May et al. 2012). The den itself is just a temporary construction, but females tend to reuse the same area in following years (Landa et al. 1998). Moreover, the same denning area can be inherited for several generations, as wolverines are to a large extent philopatric (Chapell et al. 2004).

Each winter and spring, wardens from the State Nature Inspectorate in Norway and from the Environmental Protection Agency in Sweden, searched for natal dens in the study population, to obtain a minimum count of the number of reproductive females, which has been compared between years to produce estimates of population growth rate. Also, throughout the study period, each time a wolverine den was found, its coordinates were included in a national database (http://www.rovbase.no, http://www.rovbase.se), and the site was checked for possible new reproductions in subsequent years. Thus, the monitoring system has been each year taking advantage of the information acquired during previous winters, leading to a potential increase ability to detect the study species. Between 1996 and 2013, the number of known denning sites has increased from zero to 120.

We used data from the Scandinavian wolverine monitoring programme to parameterize our simulation model and assess the effects of knowledge accumulation on the performance of the monitoring system. As a parallel programme of scat collection and DNA-based individual identification had estimated population growth rate during the study period to be 1.04 (Flagstad et al. 2013), we used this value as simulated *λ* in the model. Also, we set the site persistency parameter *π* to 5, as den sites have been reused for reproduction in average until 5 years and up to 10 years after the first detection. Survival and reproduction probabilities were set to 0.89 and 0.63, as estimated in previous studies on the same wolverine population (Brøseth et al. 2010; Brøseth and Tovmo 2013). We did not have empirical data on the probability for a young female to inherit a den site from other individuals, so we performed the simulations using a range of values between 0.2 and 0.8 for the parameter *h*. We also simulated a range of detection probabilities between 0.1 and 0.9. After running simulations, we compared the resulting estimates of population growth rate with the actual values provided by the monitoring system and assessed for which value of the detection probability parameter the two sets of estimates were more consistent.

## Results

**Figure 2:**
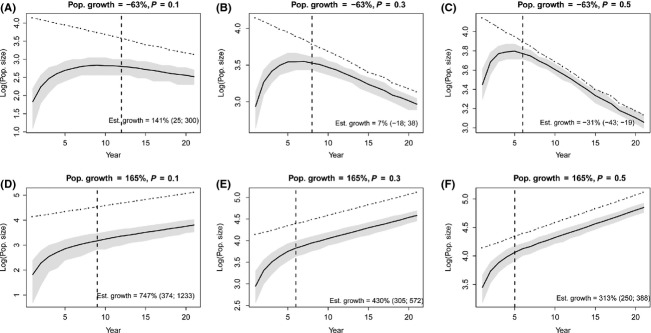
Relationship between real and estimated population trend, when using a count-based index, under different scenarios of population growth rate and detection probability. The relationship is shown for a population with *λ* = 0.95 (A–C) and *λ* = 1.05 (D–F). The dashed vertical line represents the length of the learning phase, that is the number of years necessary to obtain yearly estimates of population growth rate with bias <0.05, whereas the numbers in parentheses are 95% CIs of the estimated population growth rate. All scenarios were run for an initial population size of 500 individual, a site persistence *π* = 5 years, a probability to inherit a reproduction site *h* = 0.2, and a survival rate *ϕ* = 0.8.

When simulating a declining population (*λ* = 0.95), the effect of the learning process on the accuracy of population growth rate estimates was a high degree of overestimation. Despite the strong population decline over time (63% reduction in population size in 20 years), the simulated monitoring system erroneously produced the image of an increasing population size for several years after the beginning of the monitoring process (Fig. 2), due to the fact that the count process was influenced both by the decreasing trend in population size and the increasing trend in the ability to detect the study species. The length of the learning phase, which in this case corresponded to the time necessary to detect population decline, varied as a function of detection probability, and ranged from six (Fig. 2C) to 13 years (Fig. 2A).

We observed a similar, but less pronounced, degree of overestimation in population growth rate when simulating an expanding population (*λ* = 1.05, see Fig 2D–F). As in the previous scenario, the bias in estimating *λ* was higher at the beginning of the monitoring period, when the system was rapidly accumulating knowledge about the spatial distribution of reproduction sites, but it performed better and better with each time step. Also in this case, the total bias in population growth rate over the 20 time steps and the length of the learning phase were modulated by detection probability. Still, even for high values of this parameter (*P* = 0.5, Fig 2F), the estimated population growth rate was almost three times higher than the true one, and five years were necessary before the monitoring system started producing estimates of *λ* with an acceptable degree of bias.

When applying the count-based index to a population with a stochastic growth rate, the general patterns of the system were similar: the estimator still showed an initial learning phase in all simulated scenarios (Fig. S1 in Supporting Information), and the introduction of an environmental stochasticity factor did not affect the length of such learning phase, which was not different from the one observed under a deterministic model for the same set of input parameters. Still, increasing levels of stochasticity in the demographic model increased the unpredictability of the system on a year-to-year basis, as shown by the larger uncertainty buffers around the average population size estimates (Fig. S1).

In a second set of simulated scenarios, we compared three monitoring systems with the same detection probability (*P* = 0.4), population size and trend (*λ* = 0.95), but with a different ability to improve monitoring performance, based on the information acquired during previous years. When the parameters *π* and *h* were set to zero, corresponding to no temporal autocorrelation in the spatial distribution of reproduction site and to a monitoring system with no learning ability, population growth rate estimates were unbiased for the whole duration of the monitoring period, so that the length of the learning phase was zero (Fig. S2a in Supporting Information). When introducing an increasing potential for the monitoring system to learn from its past experiences, by manipulating the parameters *π* and *h* (Fig. S2b,c) and increasing the probability of reuse of reproduction sites, the behavior of the system suddenly changed, leading to a high overestimation of population growth rate. During the first years of monitoring, when the simulated population was decreasing by 5% each year, the monitoring system produced an impression of a population increasing by up to 30% each year, confounding the trend in the biological process with the trend in the detection process. Only after a number of years, ranging from 6 to 8 in our simulated scenarios, did the system start producing estimates of *λ* with an acceptable degree of overestimation. The comparison of the three scenarios (Fig. S2) showed that the higher the persistence of reproduction sites and the higher the probability for an individual to inherit a previously occupied site, the greater the resulting bias in the estimation of population growth rate.

**Figure 3:**
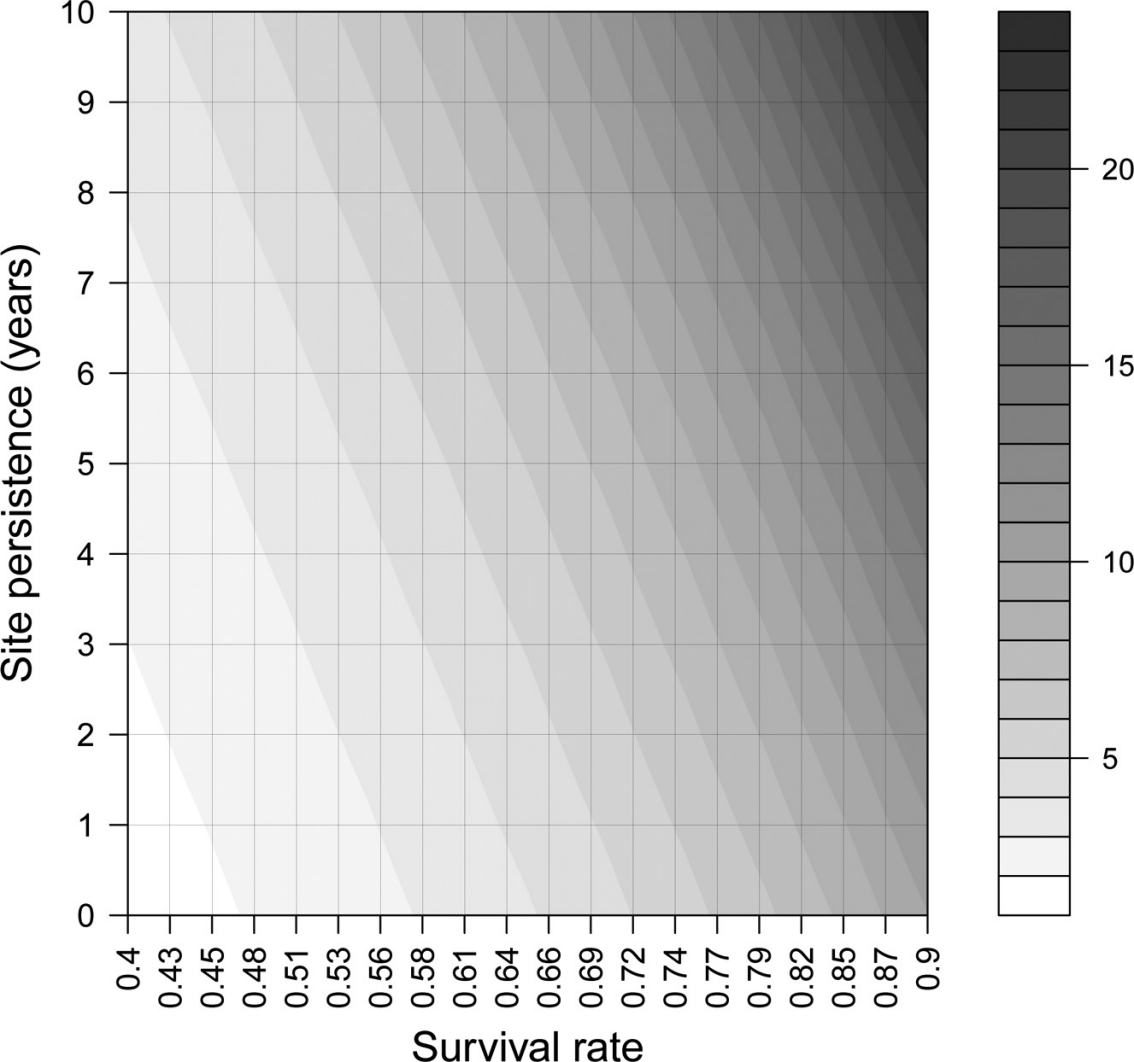
Relationship between site persistence, survival probability, and the length of the learning phase, based on the *β* coefficients shown in Table 2, and using a detection probability *P* = 0.3. Lighter to darker shades of gray indicate the predicted length of the learning phase for species with progressively higher survival rates, for a given value of the site persistence parameter *π*.

**Figure 4:**
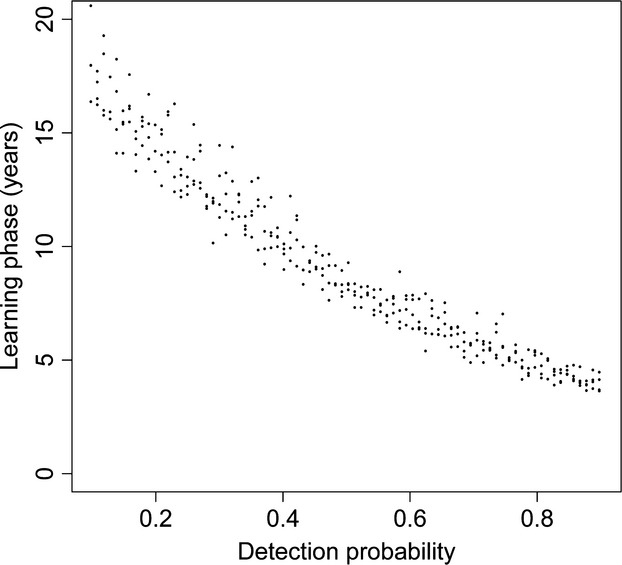
Relationship between detection probability and the length of the learning phase (the number of years necessary to obtain *λ* estimates with bias <0.05), when using a count-based index to monitor population growth rate. Predictions are based on the *β*-coefficients shown in Table 2, based on a yearly survival *ϕ* = 0.6 and a site persistence *π* = 10 years.

When allowing all simulation parameters to vary randomly and simultaneously, the sensitivity analysis showed that the length of the learning phase was primarily affected by detection probability, by survival probability, and by the persistence of reproduction sites (Table 2), with a minor support provided to the effect of reproduction and inheritance probabilities, whereas the influence of population growth rate on the length of the learning phase was negligible. Individual survival was positively correlated with the time necessary for the monitoring system to start producing unbiased estimates of population growth rate (*β* = 0.240, SE = 0.038, *P* < 0.001). The same monitoring design, with the same detection probability, was associated on average with a 10-year longer learning phase when applied to a long-lived species (*ϕ* = 0.9) than when applied to a short-lived species (*ϕ* = 0.5, see Fig. 3), as the slower turnover of individuals in a species with high survival allowed the monitoring system to accumulate and take advantage of a greater amount of information about the spatial distribution of reproduction sites. Similarly, reproduction sites with a longer persistence lead to an increase in the number of initial years during which the monitoring system systematically overestimated population growth rate (*β* = 0.111, SE = 0.038, *P* < 0.001). Also, the duration of the learning phase was strongly mediated by detection probability (*β* = −0.450, SE = 0.039, *P* < 0.001). When simulating a monitoring system with a low associated detection probability (*P* < 0.2), up to 20 years were necessary before starting to produce estimates of population trend with an acceptable level of bias (Fig. 4), whereas the time interval was reduced to <10 years when detection increased to 0.7–0.8. It should be noted, though, that all simulated monitoring systems with some degree of temporal autocorrelation in the spatial distribution of reproduction sites (*π* and *h* > 0) produced a predicted initial phase of poor monitoring performance, regardless of the associated detection probability. The complete results of the sensitivity analysis, with the regression coefficients and all input parameters, are shown in Table 2.

**Table 2:** Results of the perturbation analysis, showing the relative importance of each of the input parameters in affecting the length of the learning phase (time necessary to achieve a bias <0.05) when using a count-based index to monitor population growth rate

Parameter	Standardized *β*-coefficient	SE	*P*
Site persistence (*π*)	0.111	0.0387	<0.001
Survival rate (*ϕ*)	0.240	0.0385	<0.001
Inheritance probability (*h*)	0.027	0.0382	0.479
Detection probability (*P*)	−0.450	0.0396	<0.001
Fecundity (*f)*	−0.009	0.0380	0.809
Population growth rate (*λ*)	−0.010	0.0376	0.792

### The wolverine study case

**Figure 5:**
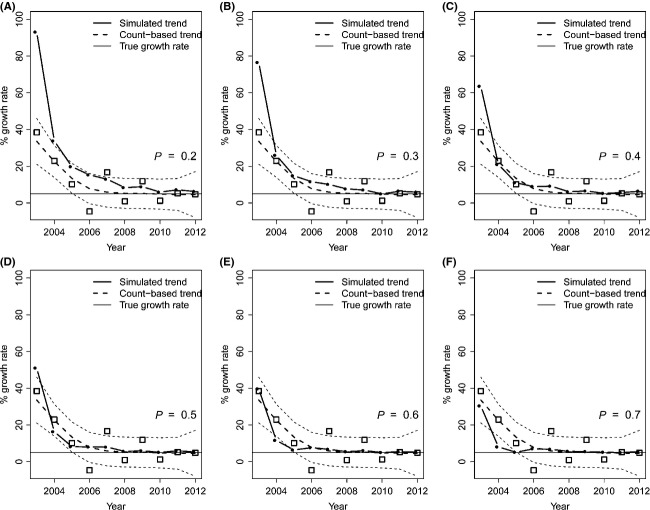
Relationship between real and estimated population trend, when parameterizing the simulation model with data from the wolverine demographic monitoring in southern Scandinavia. The relationship is shown for detection probability ranging from 0.2 to 0.7. All scenarios were run for an initial population size of 170 individual, a site persistence *π* = 5 years, survival rate *ϕ* = 0.89, and reproduction probability *f* = 0.63. Squares are population growth rate estimates provided by the monitoring system, and black dots are the estimates provided by the simulation model. The horizontal continuous line is the real growth rate (*λ* = 1.01).

**Figure 6:**
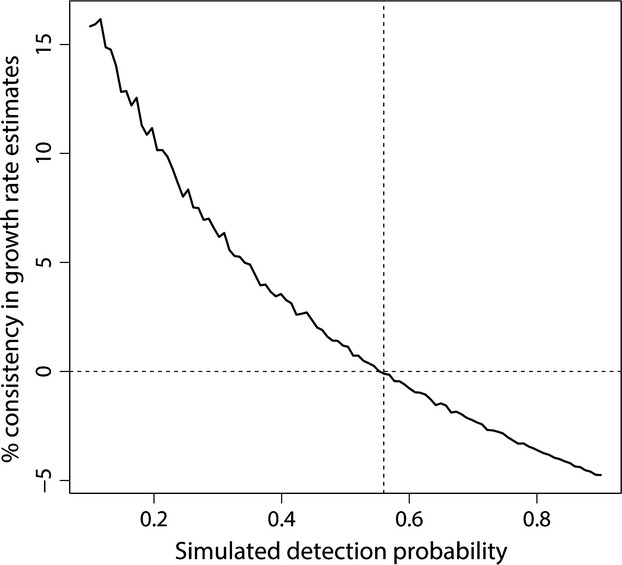
Consistency between simulated and real estimates of population growth rate for wolverines in southern Scandinavia, 2002–2013, as a function of the average level of detection probability. The vertical dashed lines, corresponding to *P* = 0.56, indicate the value of detection probability for which real and simulated estimates show the highest consistence.

When parameterizing the model with data derived from the wolverine monitoring in southern Scandinavia, population growth rate estimates were always positively biased during the first years of monitoring (Fig. 5). Within the range of parameters used for this set of simulations, the inheritance probability *h* had no substantial effect on model results. As expected, the extent of bias was dependent on the level of detection probability, and the highest fit between simulated and real population growth rate estimates was obtained for *P* = 0.56 (Fig. 6). Model results corresponding to this scenario showed that the estimates of up to a 40% increase in population size in a single year, provided by the monitoring system during the first years, were far from reality and due to the confounding effect of the increasing trend in detection probability. The Scandinavian monitoring system has been producing biased estimates of population growth rate at least until 2005.

## Discussion

Although monitoring the fluctuations in the size of wild populations is crucial in conservation and management, it is also a challenging task (Yoccoz et al. 2001), whose performance can be affected by population characteristics, sampling effort and design, environmental and demographic stochasticity, etc. (Lande et al. 2003; Nuno et al. 2013). While the implementation of systematic sampling and a formal treatment of detection probability are the best available options for this purpose from a theoretical perspective, it is still likely that for the foreseeable future, a large number of these populations will be monitored using cheaper, but less robust, indices of population trend. This becomes especially true in a period in which citizen science, that is the involvement of citizens from the nonscientific community in academic research, has become increasingly important in population monitoring and in conservation science (Dickinson et al. 2010). Citizen science does and will continue to provide demographers with a large amount of opportunistic, unstandardized monitoring data, which needs to be properly treated to avoid producing flawed conclusions about population processes (Bird et al. 2013; Tulloch et al. 2013).

The results of our individual-based simulation models show that under certain conditions, that is, when a population process retains a certain degree of temporal autocorrelation, a monitoring system is intrinsically prone to increase its knowledge about such a process with time, even if no intensification of effort and no change in the monitoring conditions occur. We show that such an increase in the accumulated knowledge about the population process can produce severe bias in the estimation of the direction and strength of the process itself, with a potentially negative impact on the ability of managers to effectively manage and preserve natural populations.

The main outcome of comparing true and estimated population trends under a wide range of demographic and monitoring parameters was a systematic tendency for the monitoring system to overestimate population growth rate. Such overestimation was stronger in the initial phase of the monitoring, when the real population process was confounded with the intrinsic accumulation of knowledge about the spatial distribution of sampling sites. As the bias in estimating *λ* was positive under all simulated conditions, the potential risk associated with the use of count-based indices seems to be especially serious for small and endangered populations, as a long time lag can occur between the start of a population decline and the detection of such a decline by the monitoring system. If we imagine that the population trend in Fig. 2B might refer to the monitoring of a small population threatened with extinction risk, such a population would have already declined by 30%, and 7 years would have passed with the illusion of an ongoing recovery, before the first negative estimate of population growth rate was provided by the monitoring system. Still after 20 years, one would have the illusion that the abundance did not decline as the monitoring programme was established, while the true abundance had declined by 63%.

Even though less pronounced, a positive bias in the estimation of population growth rate also emerged when simulating an increasing population size with time. Under this scenario, the count-based index of population growth rate always showed poor performance during the initial phase of the monitoring period, producing up to 500% positively biased estimates of *λ*. It became progressively more reliable with time, depending on the level of detection probability and the degree of persistence in the spatial distribution of sampling sites. Such a scenario of positive population trend directly applies to the actual case of several rare and elusive species in Europe and North America, such as wolves (Musiani et al. 2009), brown bears (Swenson et al. 2011), lynx (Linnell et al. 2009), and cougars (LaRue et al. 2012), which are recovering their numbers and historic distributions after being almost eradicated from their historical ranges. Also, invasive species, such as the American mink *Mustela vison* (Bonesi and Palazon 2007) and the gray squirrel *Sciurus carolinensis* (Bertolino and Genovesi 2003) in Europe, often exhibit a rapid increase in both their distribution and numerical consistency after their first settlement. Considering that both these ecological processes are often associated with high social and/or economic conflicts (Treves and Karanth 2003; Pejchar and Mooney 2009), the risk of overestimating the numerical increase of an expanding population can be serious, as it can reduce the acceptance of the species in areas where it was previously absent, and can ultimately result in higher social and economic costs for its management and conservation.

Despite our effort to allow a high degree of dynamism in the individual-based model through the inclusion of several parameters which increased the temporal turnover of reproduction sites, our model is bound to be less complex and dynamic than reality. Immigration, emigration, the geographic expansion or contraction of a species range, and changes in the patterns of habitat use are all factors contributing to modify the spatial distribution of the study species and the degree of autocorrelation in such a distribution. Therefore, even though predictions from our model showed a substantial fit between the population process and the estimated population trend after the end of an initial learning phase, reality might be more complex and less reassuring. In the model, a good performance of the monitoring system was reached when most of the used reproduction sites were sampled at least once, thus allowing the monitoring system to track future changes in population size with a good accuracy. In reality, the complex dynamics involved in a species’ demography, habitat use, selection of reproductive sites, etc., might prolong the learning phase to a much longer time than that predicted by the model, and theoretically force the monitoring system to continuously acquire new knowledge about the spatial distribution of a population process that constantly changes with time. Different types of trend curves (linear, exponential, quadratic), often resulting from different levels of human pressure and management strategies (Di Fonzo et al. 2013), can also potentially change the speed of the learning process by the monitoring system and thus the accuracy of population growth rates over time. In addition, both environmental and demographic stochasticity can increase the degree of year-to-year fluctuations in the main demographic parameters, such as survival or fertility, thus affecting the level of temporal correlation in population counts. The emergence of density dependence in vital rates could also further weaken the link between the observed counts and the underlying population process. While a more complex, spatially explicit model might provide insights to this issue, caution should always be used when interpreting the results of a count-based index of population growth rate, regardless of the number of years as the monitoring has started.

Therefore, as suggested by general theory and supported by the specific results of our work, we recommend that a systematic sampling of the population process under study and an explicit treatment of the underlying detection process should be implemented whenever economic and logistical constraints permit, as failure to include detection probability in the estimation of population growth rate can lead to serious bias and severe consequences for management and conservation. A series of analytical tools are available to produce unbiased estimates of population size and trend from simple count data, accounting for imperfect detection and a temporal trend in detectability. These methods (Dodd and Dorazio 2004; Royle 2004) require spatial and temporal replicates within the same sampling season, but with no need for individual identification, and they should be preferred over simple indices of population trend, whenever a design suitable for their application can be adopted.

The demography, spatial behavior, and expected performance of the sampling protocol in terms of detection probability should be carefully evaluated before starting a monitoring project that uses a count-based index of population trend, because the performance of the index in tracking the real population trend can be highly influenced by these factors, especially during the initial years. Finally, we recommend that managers be made aware of the fact that all simple count-based indices of population growth rate are at risk of overestimating growth, and of doing it to a higher extent during the initial implementation of a monitoring protocol. This underlines the importance of long-term monitoring projects, which allow a progressive reduction in the risk of flawed conclusions about the status and trend of a population, and also suggests that caution should be used about any conclusion drawn on the demographic trend of a population, whenever a simple index of population size has been used and only a few years of monitoring data are available.

## Supporting Information

**Appendix S1.** R function *sim_count*. **Figure S1.** Relationship between real and estimated population trend with environmental stochasticity. **Figure S2.** Effect of site persistence on monitoring performance.
