# Supplementary material for: The risks of learning: confounding detection and demographic trend when using count-based indices for population monitoring
Source: Ecol Evol. 2014 Dec 2;4(24):4637–48. doi: 10.1002/ece3.1258 (PMC4278816; doi:10.1002/ece3.1258)
Supplement: Supplementary file 1 [file ece30004-4637-sd1.docx]

**Appendix S1. R function "sim_count"**

The "sim_count" function runs the individual-based model on a set of input parameters provided by the user, producing estimates of population size, population count, real population growth rate and estimated population growth rate for each time step.

The function has the following syntax:

simcount(T ,lambda, phi, f, persistence, h,p )

The user should provide the following input parameters:

*T* : number of time steps

*lambda:* real population growth rate

*N*: Initial population size

*phi:* survival rate

*f:* proportion of individual reproducing at each time step

*persistence:* number of year a reproduction site can be re-occupied after the first time it has been used

*h*: probability for primiparous individuals to inherit a reproduction site previously used by another individual

*p*: detection probability

The function produces a plot with the observed and estimated population trend throughout the simulated monitoring period, and a table with real and estimated population size and growth rate values.

sim_count <- function(T, lambda, N, phi, f, persistence, h, p){

###########################################################################

###########################################################################

# T : number of time steps

# lambda: real population growth rate

# N: Initial population size

# phi: survival rate

# f: proportion of individual reproducing at each time step

# persistence: number of year a reproduction site can be re-occupied after the first time it has been used

# h: probability for primiparous individuals to inherit a reproduction site previously used by another individual

# p: detection probability

###########################################################################

###########################################################################

niter=1000

summary <- rep(0, niter)

mean_learning <- rep(0, (T-1))

mean_yearlambda <- rep(0, (T-1))

LCI_learning <- rep(0, (T-1))

UCI_learning <- rep(0, (T-1))

lambda_est <- matrix(0, nrow = niter, ncol = (T-1))

year_lambda <- matrix(0, nrow = niter, ncol = (T-1))

summary_bias <- matrix(0, nrow = niter, ncol = T)

new_summary <- matrix(0, nrow = niter, ncol = T)

summary2 <- matrix(0, nrow = niter, ncol = T)

N_mean <- rep(0, T)

LCI_mean <- rep(0, T)

UCI_mean <- rep(0, T)

new_mean <- rep(0, T)

LCI_new <- rep(0, T)

UCI_new <- rep(0, T)

for (k in 1:niter){

#################################################

# POPULATION PROCESS #

#################################################

den_life <- persistence

Ntot <- rep(0,T)

Ntot[1] <- N

for (t in 2:T){

Ntot[t] <- round(Ntot[t-1] * lambda, 0)

}

iner_prob <- h

R <- rep(0, length(lambda))

Nf <- round(Ntot,0)

Nreal <- rep(0,T)

survival <- matrix(0, nrow = sum(Ntot), ncol = T)

repro <- matrix(0, nrow = sum(Ntot), ncol = T)

dens <- matrix(0, nrow = sum(Ntot), ncol = T)

entered <- rep(0,T)

n_survived <- rep(0,(T))

n_repro <- rep(0,(T))

ind_den <- rep(0,sum(Ntot))

dead <- rep(0,0)

survival[1:Nf[1],1] <- 1

for (i in 1:Nf[1]){

repro[1:round(Nf[1]*f,0),1] <- 1

}

entered[1] <- Nf[1]

dens[which(repro[,1]==1),1] <- 1

ind_den[which(repro[,1]==1)] <- which(dens[,1]==1)

N_detected <- matrix(0, nrow = nrow(dens), ncol = T)

p_detection <- p

N_est <- rep(0, T)

# DETECTION PROCESS THE FIRST YEAR (NO KNOWN DENS)

for (i in 1: nrow(dens)){

N_detected[i,1] <- dens[i,1]*rbinom(1,1,prob = p_detection)

}

for (t in 2:T){

n_survived[t] <- round(sum(survival[,(t-1)])*phi,0)

survival[sample(which(survival[,(t-1)]==1),n_survived[t]),t] <-1

dead[(length(dead)+1):(length(dead)+length(which(survival[,(t-1)]==1 & survival[,t]==0)))] <- which(survival[,(t-1)]==1 & survival[,t]==0)

R[t] <- Nf[t] - n_survived[t]

entered[t] <- entered[t-1] + R[t]

survival[(entered[t-1]+1):entered[t],t] <- 1

n_repro[t] <- round(sum(survival[,t])*f,0)

repro[sample(which(survival[,t]==1),n_repro[t]),t] <-1

for (i in which(repro[,t] == 1)){

if (ind_den[i]!=0 ){dens[ind_den[i],t] <- 1}

if (ind_den[i]==0){inherit <- rbinom(1,1,iner_prob)

if(inherit == 0){dens[i,t] <- 1

ind_den[i] <- i }

if(inherit == 1 & sum(dead!=999)>0){inherited_den <- sample(dead[dead!=999],size=1)

dens[inherited_den,t] <- 1

ind_den[i] <- inherited_den

dead[dead==inherited_den] <- 999 }

if(inherit == 1 & sum(dead!=999)==0){dens[i,t] <- 1

ind_den[i] <- i }

}

}

for (i in 1: nrow(dens)){

if(sum(N_detected[i,(1:(t-1))]) > 0){

if ((t- max(which(dens[i,1:(t-1)]==1)))<=den_life){N_detected[i,t] <- dens[i,t]}

if ((t- max(which(dens[i,1:(t-1)]==1)))>den_life){N_detected[i,t] <- dens[i,t] *rbinom(1,1,prob = p_detection)}

}

if(sum(N_detected[i,(1:(t-1))]) == 0) {N_detected[i,t] <- dens[i,t] *rbinom(1,1,prob = p_detection) }

}

}

for (t in 1:T){

Nreal[t] <- sum(dens[,t])

}

# GENERATE POPULATION SIZE AND LAMBDA ESTIMATES EACH YEAR

for (t in 1: T){

N_est[t] <- sum(N_detected[,t])

}

for (t in 1: (T-1)){

lambda_est[k,t] <- ((N_est[t+1]/N_est[t]) - lambda)

}

for (t in 1: (T-1)){

year_lambda[k,t] <- (N_est[t+1]/N_est[t])

}

# CALCULATE MEAN LAMBDA ESTIMATE

summary[k] <- -(N_est[1] - N_est[T]) /N_est[1]

summary2[k,] <- N_est

} #k

# SUMMARIZE RESULTS WHEN ALL ITERATIONS ARE DONE

for(t in 1:T){

N_mean[t] <- mean(summary2[,t], na.rm=T)

LCI_mean[t] <- quantile(sort(summary2[,t]),probs = c(0.025,0.975),na.rm=T)[1]

UCI_mean[t] <- quantile(sort(summary2[,t]),probs = c(0.025,0.975),na.rm=T)[2]

}

summary[summary=="Inf"] <- NA

lambda_est[lambda_est=="Inf"] <- NA

mean_lambda_est <- mean(summary, na.rm=T)

LCI_lambda_est <- quantile(sort(summary),probs = c(0.025,0.975), na.rm=T)[1]

UCI_lambda_est <- quantile(sort(summary),probs = c(0.025,0.975), na.rm=T)[2]

for (ii in 1:ncol(lambda_est)){

mean_yearlambda[ii] <- mean(year_lambda[,ii], na.rm=T)

mean_learning[ii] <- mean(lambda_est[,ii], na.rm=T)

LCI_learning[ii] <- quantile(sort(lambda_est[,ii]),probs = c(0.025,0.975), na.rm=T)[1]

UCI_learning[ii] <- quantile(sort(lambda_est[,ii]),probs = c(0.025,0.975), na.rm=T)[2]

}

learning2 <- min(which(mean_learning<0.05))

plot(1:T, (Ntot), type = "l",ylim =c(min((LCI_mean)),max((Ntot))), lwd = 2,cex.axis = 1.5, cex.lab = 1.5, xlab = "Year", ylab = "Pop. size / Pop. count")

polygon(x=c(1:T, T:1), y=c((LCI_mean), (UCI_mean[T:1])), col="gray90", border="gray90")

lines(1:T, (N_mean), type = "l", lwd = 2)

abline (v = (learning2+1), lwd=2, lty = 2)

output <- matrix(NA,nrow=4, ncol=T)

rownames(output) <- c("Pop. size", "Pop. count", "Real lambda","Estimated lambda")

colnames(output) <- 1:(T)

output[1,] <- round(Ntot,0)

output[2,] <- N_mean

output[3,2:T] <- lambda

output[4,2:T] <- round(mean_yearlambda,2)

output

}


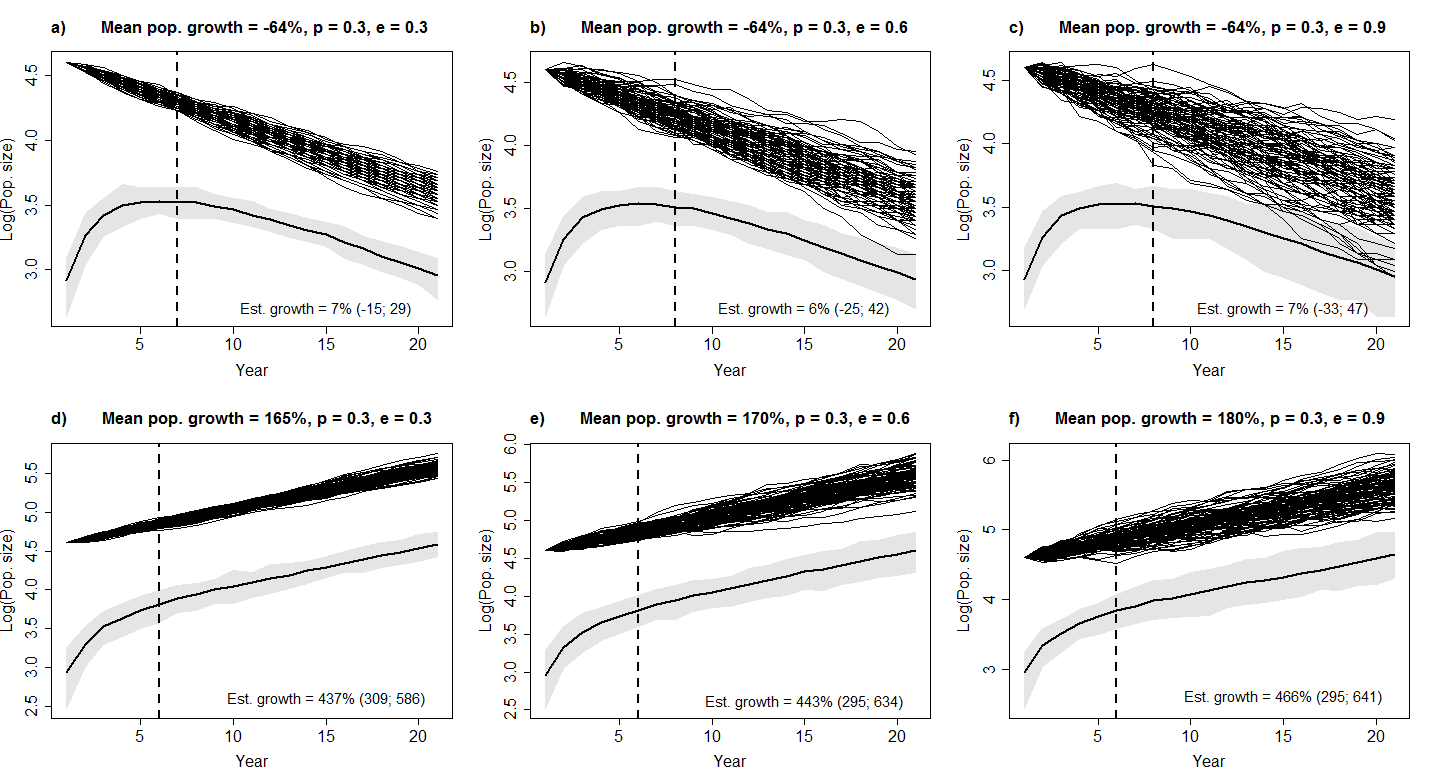


Fig. S1 - Relationship between real and estimated population trend, when using a count-based index, under different scenarios of population growth rate and environmental stochasticity. The relationship is shown for a population with an average λ = 0.95 (a, b, c) and1.05 (d, e, f), and the CV of λ (parameter *e*) ranging from 0.3 to 0.9. The dashed vertical line represents the length of the learngin phase, i.e. the number of years necessary to obtain yearly estimates of population growth rate with bias < 0.05, whereas the numbers in parentheses are 95% CIs of the estimated population growth rate. All scenarios were run for an initial population size of 500 individual, a site persistence π = 5 years, a probability to inherit a reproduciton site h = 0.2, and a survival rate φ = 0.8.


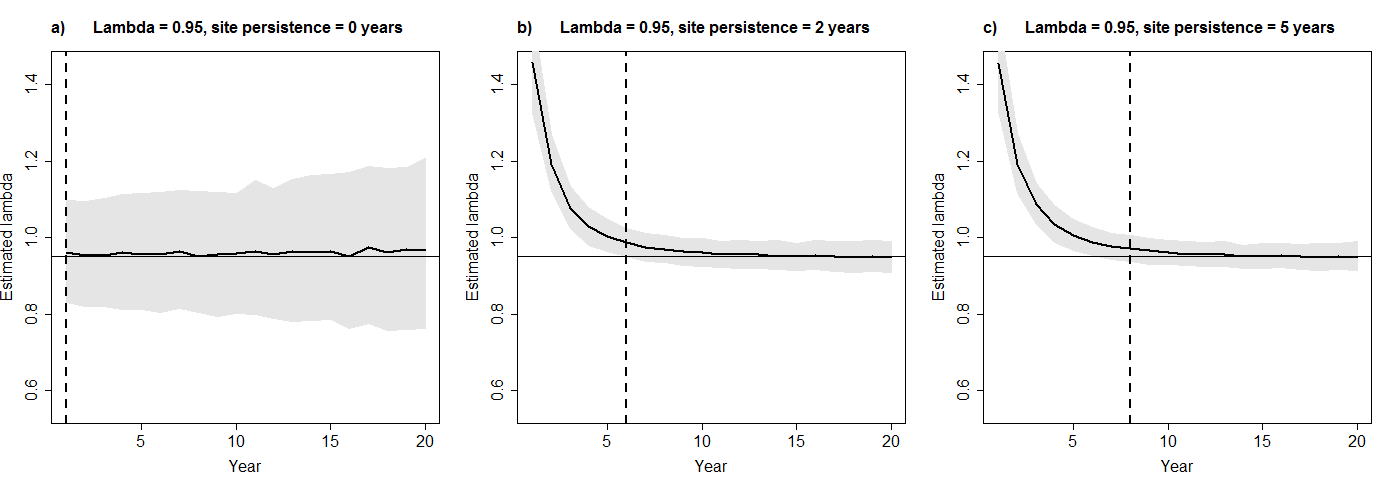


Fig. S2 - Performance of a count-based index of population growth rate when applied to species with a different persistence of reproduction sites, and a different individual probability to inherit a reproduction site already used by others in the past. Fig. 4a simulates a scenario with no temporal correlation in the spatial distribution of reproduction sites, whereas Figs. 4b and 4c simulate two increasing levels of correlation. The dashed vertical line represents the length of the learning phase, i.e. the number of years necessary to obtain yearly estimates of population growth rate with bias < 0.05. All scenarios were run for an initial population size of 500 individual, a population growth rate λ = 0.95, a detection probability p = 0.4, and a survival rate φ = 0.8.
